# Automatic Lung Segmentation and Quantification of Aeration in Computed Tomography of the Chest Using 3D Transfer Learning

**DOI:** 10.3389/fphys.2021.725865

**Published:** 2022-02-04

**Authors:** Lorenzo Maiello, Lorenzo Ball, Marco Micali, Francesca Iannuzzi, Nico Scherf, Ralf-Thorsten Hoffmann, Marcelo Gama de Abreu, Paolo Pelosi, Robert Huhle

**Affiliations:** ^1^Pulmonary Engineering Group, Department of Anaesthesiology and Intensive Care Therapy, University Hospital Carl Gustav Carus, Technische Universität Dresden, Dresden, Germany; ^2^Department of Surgical Sciences and Integrated Diagnostics, IRCCS AOU San Martino IST, University of Genoa, Genoa, Italy; ^3^Max Planck Institute for Human Cognitive and Brain Sciences, Leipzig, Germany; ^4^Department of Diagnostic and Interventional Radiology, University Hospital Carl Gustav Dresden, Technische Universität Dresden, Dresden, Germany; ^5^Department of Intensive Care and Resuscitation, Anesthesiology Institute, Cleveland Clinic, Cleveland, OH, United States; ^6^Department of Outcomes Research, Anesthesiology Institute, Cleveland Clinic, Cleveland, OH, United States

**Keywords:** uNet, COVID-19, lung segmentation, ARDS, Jaccard index, deep learning, transfer learning, lung recruitment

## Abstract

**Background:**

Identification of lung parenchyma on computer tomographic (CT) scans in the research setting is done semi-automatically and requires cumbersome manual correction. This is especially true in pathological conditions, hindering the clinical application of aeration compartment (AC) analysis. Deep learning based algorithms have lately been shown to be reliable and time-efficient in segmenting pathologic lungs. In this contribution, we thus propose a novel 3D transfer learning based approach to quantify lung volumes, aeration compartments and lung recruitability.

**Methods:**

Two convolutional neural networks developed for biomedical image segmentation (uNet), with different resolutions and fields of view, were implemented using Matlab. Training and evaluation was done on 180 scans of 18 pigs in experimental ARDS (*u*2*Net*_*Pig*_) and on a clinical data set of 150 scans from 58 ICU patients with lung conditions varying from healthy, to COPD, to ARDS and COVID-19 (*u*2*Net*_*Human*_). One manual segmentations (MS) was available for each scan, being a consensus by two experts. Transfer learning was then applied to train *u*2*Net*_*Pig*_ on the clinical data set generating *u*2*Net*_*Transfer*_. General segmentation quality was quantified using the Jaccard index (*JI*) and the Boundary Function score (*BF*). The slope between *JI* or *BF* and relative volume of non-aerated compartment (*S*_*JI*_ and *S*_*BF*_, respectively) was calculated over data sets to assess robustness toward non-aerated lung regions. Additionally, the relative volume of ACs and lung volumes (LV) were compared between automatic and MS.

**Results:**

On the experimental data set, *u*2*Net*_*Pig*_ resulted in *JI* = 0.892 [0.88 : 091] (median [inter-quartile range]), *BF* = 0.995 [0.98 : 1.0] and slopes *S*_*JI*_ = −0.2 {95% conf. int. −0.23 : −0.16} and *S*_*BF*_ = −0.1 {−0.5 : −0.06}. *u*2*Net*_*Human*_ showed similar performance compared to *u*2*Net*_*Pig*_ in *JI*, *BF* but with reduced robustness *S*_*JI*_ = −0.29 {−0.36 : −0.22} and *S*_*BF*_ = −0.43 {−0.54 : −0.31}. Transfer learning improved overall *JI* = 0.92 [0.88 : 0.94], *P* < 0.001, but reduced robustness *S*_*JI*_ = −0.46 {−0.52 : −0.40}, and affected neither *BF* = 0.96 [0.91 : 0.98] nor *S*_*BF*_ = −0.48 {−0.59 : −0.36}. *u*2*Net*_*Transfer*_ improved *JI* compared to *u*2*Net*_*Human*_ in segmenting healthy (*P* = 0.008), ARDS (*P* < 0.001) and COPD (*P* = 0.004) patients but not in COVID-19 patients (*P* = 0.298). ACs and LV determined using *u*2*Net*_*Transfer*_ segmentations exhibited < 5% volume difference compared to MS.

**Conclusion:**

Compared to manual segmentations, automatic uNet based 3D lung segmentation provides acceptable quality for both clinical and scientific purposes in the quantification of lung volumes, aeration compartments, and recruitability.

## 1. Introduction

The ongoing COVID-19 pandemic has focused attention on Acute Lung Injury and the Acute Respiratory Distress Syndrome (ARDS), a disease mainly characterized by impaired gas exchange driven by an inflammatory state of the lung (Ferguson et al., 2012; The ARDS Definition Task Force*, 2012). Optimal treatment of this pathology is currently being debated and different approaches have been proposed (Amato et al., 2009; Calfee et al., 2014; Coppola et al., 2018; Pelosi et al., 2018; Hodgson et al., 2019; Robba et al., 2020). One of the main clinical questions remaining is how to choose the best ventilator strategy.

The primary objectives of mechanical ventilation (MV) are maintaining physiological blood oxygen and carbon dioxide concentrations. However, MV itself may induce further damage to the lung parenchyma. This process is known as Ventilator Induced Lung Injury (VILI) (Slutsky, 1999; Slutsky and Ranieri, 2013). The concept of protective ventilation has thus been introduced (Network, 2009) to minimize VILI. While pathophysiological pathways leading to biotrauma (Curley et al., 2016), volutrauma (Güldner et al., 2016), barotrauma (Anzueto et al., 2004), and atelectrauma (Tsuchida et al., 2012; Güldner et al., 2016) have been identified, the clinical challenge of individual patient ventilator settings to minimize VILI still remains. The titration of ventilatory parameters is often approached by integrating functional assessments of gas exchange, mechanical properties of the lung and radiological findings. Both gas exchange parameters and lung mechanics can be reliably measured bedside, leading to useful assessments of ventilation to perfusion matching, dead space estimation, and mechanical stress on the lung. Conversely, important radiological findings such as aeration compartments and recruitability are often only assessed qualitatively. Clinicians often rely on all of these sources of information in deciding to perform interventions such as positive end-expiratory pressure (PEEP) setting, recruitment maneuvers, prone positioning, pharmacologic interventions, or extra-corporeal circulation (Battaglini et al., 2021).

The classification and quantification of lung regions on computer tomographic (CT) data may also be used to guide ventilatory strategies (Pelosi et al., 2011; Cereda et al., 2019; Robba et al., 2020). While often used in research settings (Ball et al., 2017), this is, however, not routinely performed in clinical settings since it requires costly manual lung segmentation by trained physicians. The challenge of segmenting pathologic lung parenchyma originates from the fact that non-aerated lung tissue is not distinguishable from nearby structures by either its Hounsfield unit nor by its pattern. Segmenting lung parenchyma thus requires knowledge regarding the anatomical boundary and shape of the lung. For this reason the several deterministic algorithms previously proposed (Hu et al., 2001; Karmrodt et al., 2006; Cuevas et al., 2009; Mansoor et al., 2014; Noshadi et al., 2017) either lack in accuracy or are prone to fail if any one of their numerous constituting assumptions is not met, which typically occurs in ARDS.

The segmentation challenge posed by pathologic lung parenchyma has been recently successfully tackled using artificial intelligence (AI) based algorithms such as convolutional neural networks (CNN) (Shelhamer et al., 2017). The SegNet (Badrinarayanan et al., 2017) architecture was used successfully for the automatic segmentation of healthy and injured lung scans from experimental and clinical data alike (Gerard et al., 2020). More recently polymorphism was added, further increasing the robustness of the algorithm in segmenting poorly or non-aerated lung regions on CT scans with up to 25% volume of the non-aerated lung compartment (Gerard et al., 2021). Such U-net like architectures constitute an improvement compared to previous CNNs, mainly in context feedback. These architectures are thus particularly well suited to scarce segmentation problems with only limited available data (Ronneberger et al., 2015). For example, U-nets have been applied to medical image recognition and tasks such as brain tumor segmentation (Çiçek et al., 2016). When applied to the task of lung parenchyma segmentation, U-nets have shown promising results on healthy chest CTs by Ait Skourt et al. (2018) and on 2D slices (Zhou et al., 2021) and 3D volumes (Müller et al., 2020) of COVID-19 CT scans.

Given these promising results, in this contribution we propose a three-dimensional U-net based algorithm for segmenting lungs across different pathological states. We develop our system using experimental CT data. The resulting algorithm can be run on personal computers. We further train and evaluate this algorithm on data from a cohort of ICU patients with both non-respiratory diseases and respiratory disease, including COVID-19. We perform the evaluation of the system in terms of the correct determination of aeration compartments and lung volumes.

## 2. Materials and Methods

### 2.1. Study Design

The present study is aimed at developing a reliable and time-efficient method for lung segmentation in pathological conditions using available data sets for future application. To this end, we employed only previously gathered research data sets with granted appropriate ethics committee approvals. Data had already been annonymized within the original study.

**Figure 1:**
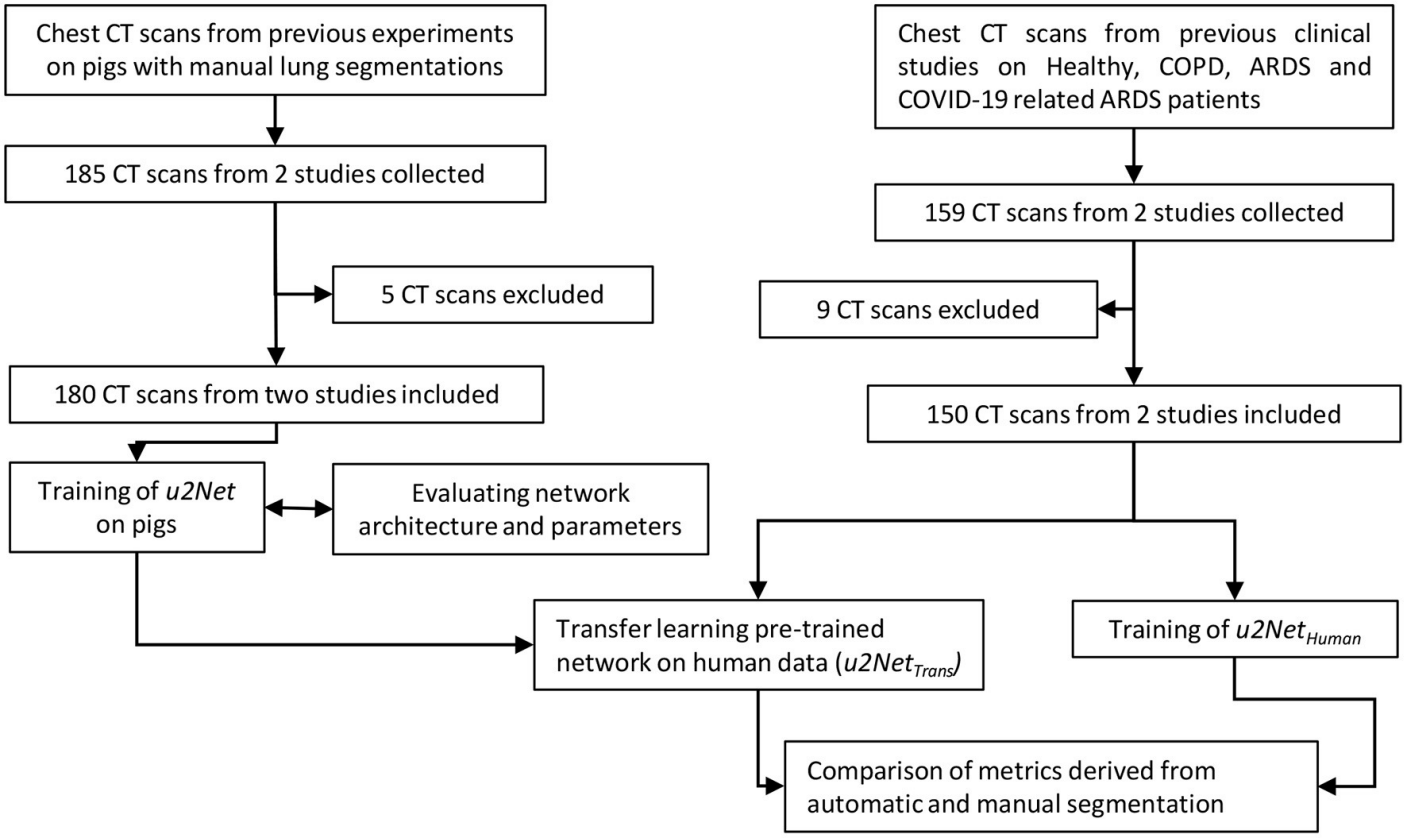
Flow chart of the design process applied (CT scans were excluded if manual segmentations were of poor quality).

The study was conducted in three phases. First, we compiled the animal data set and used it to select the better of two possible network architectures. We then used the clinical data set to test *ex-novo* training vs. transfer learning from the animal data set. Finally, we evaluated if our approach was acceptable for research and clinical applications. To do so we compared measures derived from lung CT segmentations, such as aeration compartments, effective lung volume and recruitability, as calculated from CNN-segmentations against the same measures calculated from manual CT segmentations. An outline of the process is shown in Figure 1.

### 2.2. Convolutional Neural Network

**Figure 2:**
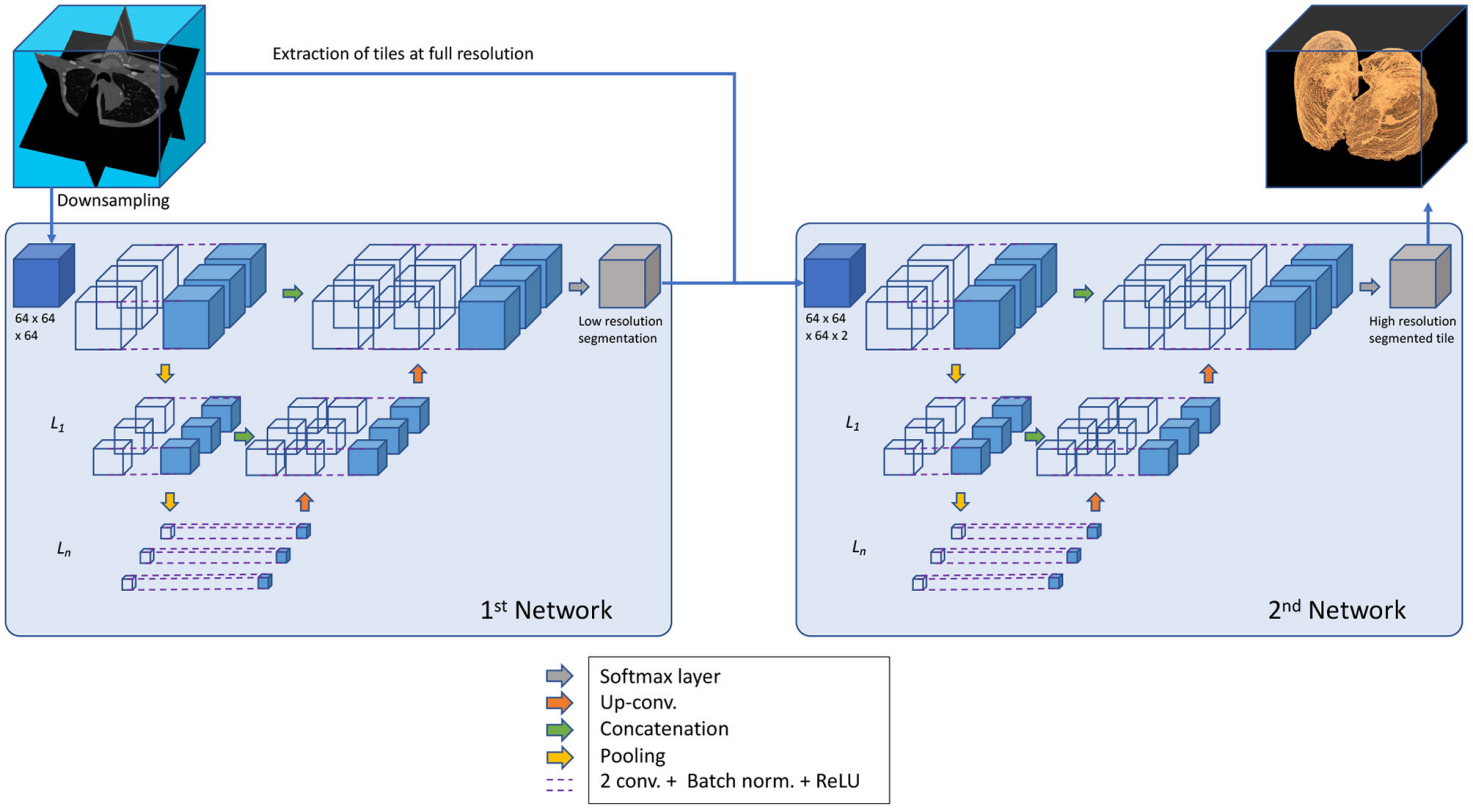
General structure of the segmentation process applied in *u*2*Net*s.

The architecture implemented here stems from U-net structures, that apply convolutions to different image resolutions. Our architecture expands the same concept to 3D volumes. U-nets use down-sampling on the encoding path of the image processing, before applying convolutions, to modify the resolution of the image itself and then implement a symmetric up-sampling and concatenation of the results before the final convolution layer (Sudre et al., 2017) (Figure 2).

This algorithm of U-nets is composed of two networks that operate in series, as suggested by Gerard et al. (2020). For the first network CT data was down-sampled to 64 × 64 × 64 voxel. This network has the task of determining general shape and size of the lung. A second network fine tunes the segmentation using as input both the output from the first network and the full-scale CT, re-sampled at one millimeter isotropic voxel for standardization across data sets. The second network operates by dividing the data into tiles that can be managed by a current desktop computer, but has only a partial view of the CT and relies on the output of the first network for information about size and shape.

Hyper parameters chosen for all networks based on previous literature were: three encoding steps, 32 first encoder filters, and 3 × 3 × 3 convolutional filters. At every encoder level convolution (Stride 1 × 1 × 1 voxel, same padding), batch normalization, and linear rectification was performed twice followed by max pooling. CT data were not augmented. However, both clinical data sets included implicit data augmentation, since scans at different resolution and different CT reconstruction kernels were used. Training was performed using an Adam solver and an initial learning rate of 10^−4^ with the DICE loss function. Weights were initialized according to He et al. (2015).

Architectures and network training were implemented in Matlab using the Deep Learning Toolbox (Mathworks Inc., Natwick, MA, USA). Training and validation were run in parallel on multiple GPUs on the High Performance Cluster (HPC) at the centre of information services and high performance computing (ZIH) at the TU-Dresden, Germany.

Two connected neuronal networks *u*2*Net*_64_ were implemented in series and the role of the second networks field of view on the transversal plane (64 × 64 × 64 voxel - *u*2*Net*_64_) or (128 × 128 × 32 voxel - *u*2*Net*_128_) was investigated on experimental data. The algorithm yielding highest performance on the experimental data was then used to perform training and evaluation on clinical data only (*u*2*Net*_*Human*_).

Finally, we tested the usefulness of transfer learning. Specifically, a u2Net with the same architecture as *u*2*Net*_*Human*_ was initially trained on the pig data set. The resulting network weights and biases were then kept constant on all layers except the final convolution and classification layers. These weights were re-trained on the human data set, with increased weight and bias learning rate factors to optimize the computational costs of training. The resulting network (*u*2*Net*_*Trans*_) was then compared to *u*2*Net*_*Human*_.

### 2.3. Data Sets

CT scans from two completed animal experimental studies and two clinical studies were used (Supplementary Table 1). One manual segmentation was available for each scan. Each manual segmentation had been performed and corrected by two experienced experts. These data were employed for the training and parametrization of the described neural network algorithms.

#### 2.3.1. Experimental Data

68 scans from 11 animals were taken from previously completed experimental study (Güldner et al., 2014). This study investigated the effects of different degrees of spontaneous breathing during biphasic positive airway pressure (BIPAP) ventilation on neutrophilic inflammation in a double-hit ARDS model composed of repeated lung lavage with Horowitz ratio below 200 *mmHg* for 30 min. CT scans were acquired using Siemens Biograph 16 Hirez PET/CT (Siemens Knoxville, TN, USA) at a resolution of 0.4*x*0.4*x*1 *mm*. Scans were taken during end-expiratory occlusion at an airway pressure of 10 *cmH*_2_*O* of 10*s*. The study protocol was approved by local animal care committee (Landesdirektion Dresden, Dresden, Germany). Further protocol details are described elsewhere (Güldner et al., 2014).

A further 112 scans from 7 animals were taken from an unpublished experimental study performed at the University Hospital Carl Gustav Carus, TU Dresden, Germany. The study was performed on non-injured pig lungs with negative end-expiratory airway pressure of as low as −12 *cmH*_2_*O*. The CT scans (Kernel: BF30f, Resolution: 0.59*x*0.59*x*3 *mm*) were acquired using SOMATOM Definition Edge (Siemens Healthineers, Erlangen, Germany) in supine position during end-expiratory and end-inspiratory hold of 10 s with a PEEP of 5 *cmH*_2_*O* as well as negative externally applied abdominal pressure (NEAP) at the airway of 0, −5, −8, and −12 *cmH*_2_*O*. The Institutional Animal Care and Welfare Committee of the State of Saxony, Germany approved all animal procedures (DD24.1-5131/474/422).

A total of 180 static CT scans from pigs were thus used for training and 5-fold cross validation as described below.

#### 2.3.2. Clinical Data

Patient CT scans were collected from previously published studies with available manual segmentations performed by expert radiologists. A total of 159 scans from healthy, COPD and ARDS patients from the University Hospital San Martino in Genoa, Italy, were included in the current study. One set of 112 scans were taken from a previous study assessing the influence of reconstruction kernels and slice thickness on the estimation of aeration compartments across pathological conditions (Ball et al., 2016) (KERNEL). A further set of 44 scans from 18 patients were taken from another previous study investigating the effects of PEEP levels (8 and 16 *cmH*_2_*O*) on alveolar recruitment in mechanically ventilated COVID-19 patients (Ball et al., 2021) (PEEP). Change of relative mass of non- and poorly aerated compartments from *PEEP* = 16 *cmH*_2_*O* to *PEEP* = 8 *cmH*_2_*O* was used to quantify recruitable lung tissue in a sub-set of 12 COVID-19 patients.

Data acquisition protocols, patient demographic data, ethics committee approval and further details can be found online in the original publications (Ball et al., 2016, 2021).

### 2.4. Five-Fold Cross Validation

Due to the relative scarcity of segmented CT scans,rather than splitting our experimental data in fixed training and validation sets, we instead employed a 5-fold cross validation procedure. This means that each network was trained five times and for each iteration 80% of available scans were randomly selected for training and the respective remaining 20% were used for validation.

### 2.5. Evaluation of Segmentation Quality

Performance of the automatic segmentation was assessed in two categories:

1. Similarity was assessed by:
  - Jaccard Index (*JI*), the ratio of number of elements of the intersection and the number of elements of the union of two sets - thus quantifying similarity - defined by
    (1) $$\begin{array}{l} JI=Jaccard\left(GT,PR\right)=\frac{\left|GT\cap PR\right|}{\left|GT\cup PR\right|} \end{array}$$
    where ground truth (*GT*) and the prediction (*PR*) correspond to logical masks (true or false) specifying whether a voxel belongs to the lung ROI or not. In our case GT corresponds to the manual segmentation. Perfect overlap between GR and PR results in a Jaccard Index of 1, whereas no intersection would result in a Jaccard Index of 0.
  - The Jaccard index is related to the popular Sørensen–Dice coefficient according to
    (2) $$\begin{array}{l} DICE=\frac{2\cdot JI}{1+JI}. \end{array}$$
    In the current study we decided to use *JI* instead of *DICE* since the former allows for a more granular analysis, especially for values close to *JI* = 1 (Supplementary Figure 1).
2. Contour agreement was assessed by:
  - Boundary Function score (BF-score) was calculated as proposed by Csurka et al. (2013). Briefly, precision and recall per class *c* are defined as:
    (3) $$\begin{array}{l} P^{c}=\frac{1}{\left|B_{PR}\right|}\underset{z\in B_{PR}^{c}}{\sum }\left[d\left(z,B_{GT}^{c}\right)<\theta \right] \end{array}$$
    and
    (4) $$\begin{array}{l} R^{c}=\frac{1}{\left|B_{GT}\right|}\underset{z\in B_{GT}^{c}}{\sum }\left[d\left(z,B_{PR}^{c}\right)<\theta \right] \end{array}$$
    with boundary map of the ground truth $B_{GT}^{c}$, boundary map of the predicted segmentation $B_{PR}^{c}$, Euclidean distance *d*, and distance error tolerance θ (chosen to be 0.75% of the image diagonal). The BF-score for class *c* is then derived by
    (5) $$\begin{array}{l} BF^{c}=\frac{2\cdot P^{c}\cdot R^{c}}{R^{c}+P^{c}} \end{array}$$
    where a perfect BF-score of 1 indicates that both segmentation boundaries are within the distance error tolerance θ of each other.
  - Average symmetric surface distance (*ASSD*) was calculated (Yeghiazaryan and Voiculescu, 2018) as:
    (6) $$\begin{array}{l} ASSD\left(B_{PR},B_{GT}\right)=\frac{1}{\left|B_{PR}|+|B_{GT}\right|}\times (\underset{x\in B_{PR}}{\sum }d_{min}\left(x,B_{GT}\right)\\ \;+\underset{y\in B_{GT}}{\sum }d_{min}\left(y,B_{PR}\right)). \end{array}$$
    A comparison of both measures *BF* and *ASSD* in an *in-silico* example may be found in the Supplementary Figure 2.

We anticipated that the segmentation quality of the proposed algorithm would depend on the degree of lung injury and, more specifically, on the size of non-aerated lung regions of the respective scan. To quantify the robustness of the segmentation method, we took the slope *S*_*JI*_ between *JI* and the relative volume of non-aerated compartments, defined by voxel value < −100 *HU* (*V*_*nA*_ in *arb*.*un*.) of the respective manually segmented region of interest (ROI) of the lung. This slope was determined by fitting the following linear equation over all scans in the respective data set:

(7) $$\begin{array}{l} JI=S_{JI}\cdot V_{nA}+C \end{array}$$

A robust segmentation algorithm should be independent of the degree of the non-aerated compartment size, thus resulting in a *S*_*JI*_ = 0(*arb*.*un*.). Any negative/positive slope would instead indicate worse/better segmentation quality for non-aerated lung regions. The slopes *S*_*DICE*_, *S*_*BF*_, and *S*_*ASSD*_ were calculated the same way and have similar interpretation.

### 2.6. Aeration Compartment Size and Effective Lung Volume

The analysis of lung aeration compartments based on CT data is performed in Matlab (Mathworks Inc., Natwick, MA, USA). We employed commonly accepted thresholds dividing segmented lungs into four compartments using Hounsfield Unit (HU) value: Hyper-aerated < −900, −900 < normally aerated < −500, −500 < poorly aerated < −100, and non-aerated > −100. The relative size *%volume* of each compartment within the automatically segmented lung ROI was compared to the one determined by manual segmentation. The effective lung volume (ELV) was determined as the gas volume within the automatically segmented lung ROI and compared to ELV as determined using the manual segmentation.

### 2.7. Statistical Analysis

Statistical analyses were performed using non-parametric Wilcoxon test and slope differences assessed by confidence intervals. Agreement between relative aeration compartment sizes computed using automatic and manual segmentations was evaluated as proposed by Bland and Altman (1986). Statistical analyses were performed using the R statistical programming language (R Core Team, 2021). Statistical significance was accepted for *P* < 0.05.

## 3. Results

### 3.1. Performance on Experimental Data Sets

The network designed with a wider transversal input *u*2*Net*_128_ outperformed the network designed with a wider longitudinal view across all quality features (Table 1). Additionally, the two network architectures did not differ in terms of robustness relative to non-aerated lung volume: slopes *S*_*JI*_ and *S*_*BF*_ did not differ between *u*2*Net*_64_ and *u*2*Net*_128_.

**Table 1:** Segmentation quality metrics for the networks *u*2*Net*_64_ and *u*2*Net*_128_ on the experimental data set.

	***u*2*Net*_64_**	***u*2*Net*_128_**	**Sign**.
*DICE* (arb. un.)	0.942 [0.93..0.95]	0.955 [0.95..0.96]	*P* < 0.001
*JI* (arb. un.)	0.891 [0.88..0.9]	0.913 [0.9..0.93]	*P* < 0.001
*BF* (arb. un.)	0.993 [0.97..1]	0.997 [0.98..1]	*P* = 0.009
*ASSD* (mm)	1.149 [1.01..1.78]	0.899 [0.79..1.25]	*P* < 0.001
*S*_*JI*_ (arb. un.)	−0.15 {−0.19..−0.12}	−0.2 {−0.23..−0.17}	
*S*_*BF*_ (arb. un.)	−0.049 {−0.09..0}	−0.082 {−0.13..−0.04}	

*values as median [iqr] and slope {95% conf. int.} respectively; with Sørensen–Dice coefficient (DICE), Jaccard index (JI), BF-score (BF), and average symmetric surface distance (ASSD) and their respective slopes S_JI_ and S_BF_; statistics according to Wilcoxon test*.

Both *u*2*Net*s slightly over-estimated relative volume of non-aerated and under-estimated relative volume of normally aerated lung regions, while relative volumes of poorly and hyper-aerated as well as *ELV* did not differ significantly (Table 2).

**Table 2:** Relative volume of aeartion compartments and effective lung volume (*ELV*) as determined using the networks lung ROI predictions *u*2*Net*_64_ and *u*2*Net*_128_ on the experimental data set.

	**Ref. mask**	***u*2*Net*_64_**	***P* =**	***u*2*Net*_128_**	***P* =**
*V*_*nA*_ (%)	12.3 ± 8.5	15.9 ± 8.0	<0.001	15.8 ± 8.0	<0.001
*V*_*poor*_ (%)	25.6 ± 8.1	25.6 ± 7.2	0.971	25.7 ± 7.2	0.952
*V*_*norm*_ (%)	56.0 ± 16.6	51.5 ± 14.5	0.007	51.7 ± 14.5	0.009
*V*_*hype*_ (%)	3.5 ± 3.6	3.4 ± 3.5	0.879	3.4 ± 3.5	0.876
*ELV* (*ml*)	757 ± 259	768 ± 262	0.879	768 ± 262	0.876

*values as mean ± sd; P-values indicate difference compared to reference mask from two sample T-test; with rel. volume of non-aerated (V_nA_), poorly-aerated (V_poor_), normally aerated (V_norm_) and hyper-aerated (V_hype_)*.

### 3.2. Performance on Clinical Data

**Figure 3:**
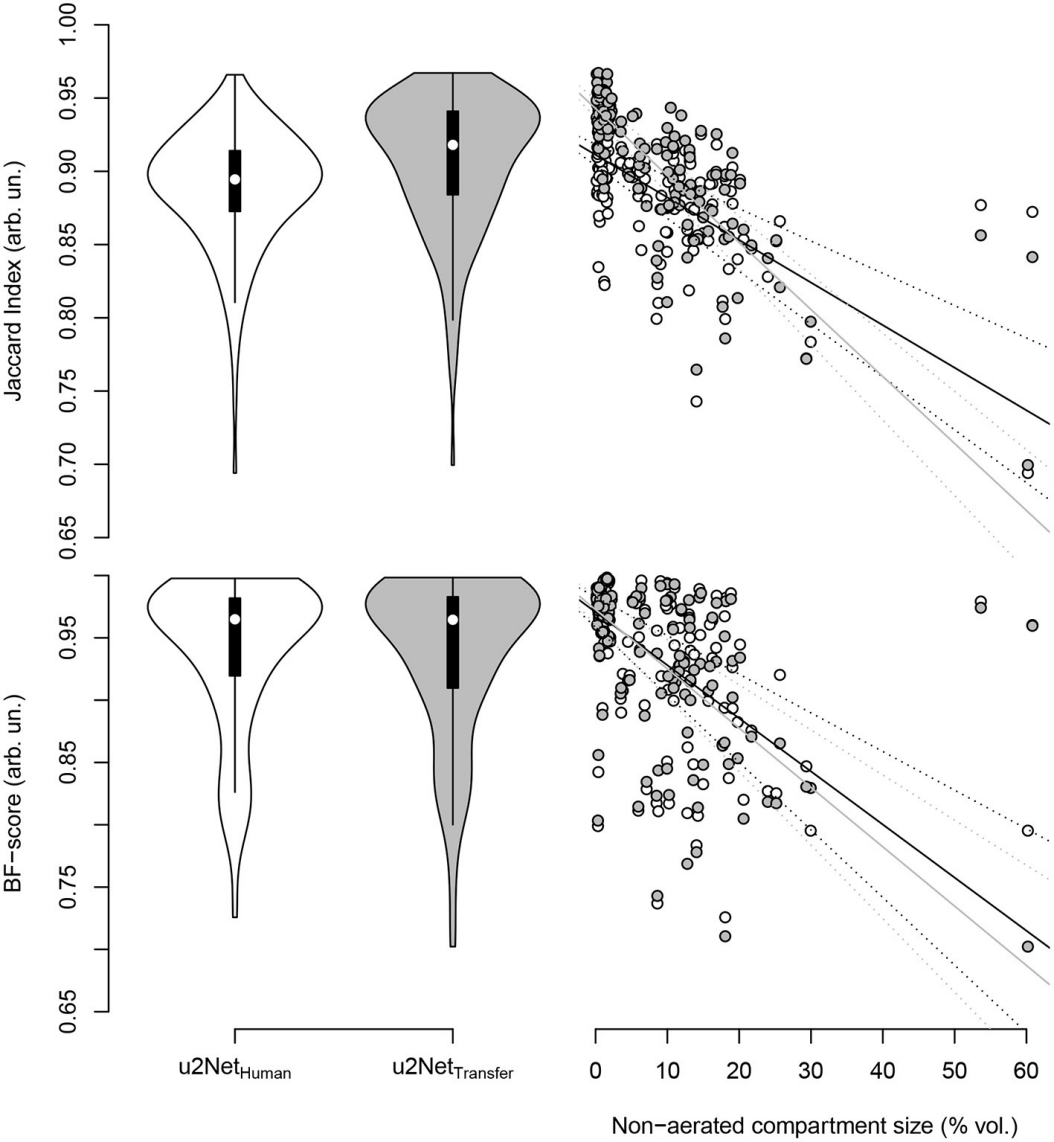
Jaccard index **(top)** and BF-score **(bottom)** for networks trained from human data only (*u*2*Net*_*Human*_, “white”) and through transfer learning of networks trained on animal CT data (*u*2*Net*_*Transfer*_, “grey.”) In the right column the respective measure over relative size of the non-aerated compartment in % volume and its linear regressions with slopes *S*_*JI*_ and *S*_*BF*_, as well as their respective confidence intervals.

**Figure 4:**
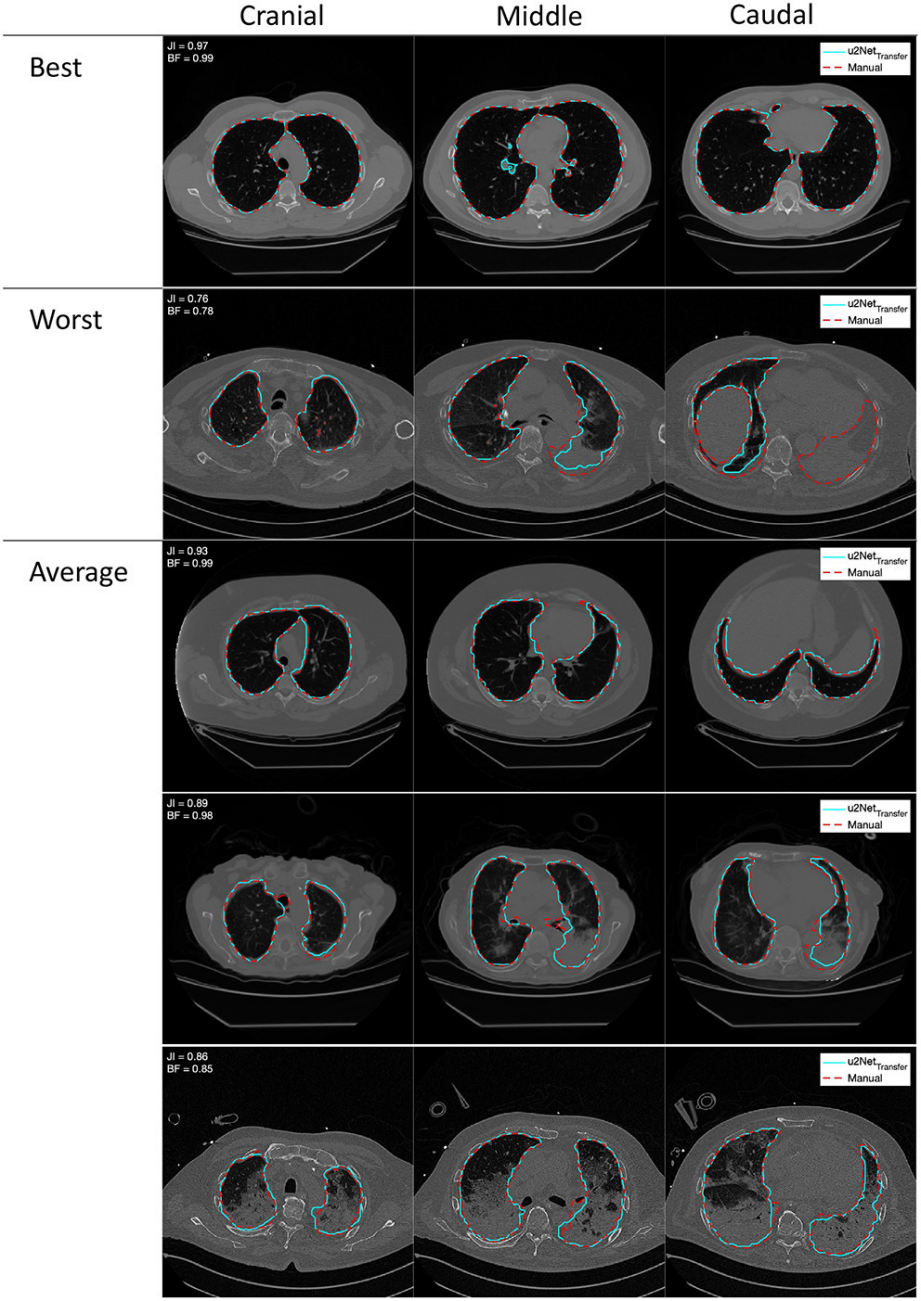
Outlines of best, worst, and average segmentations generated from the double resolution architecture *u*2*Net*_*Transfer*_ (cyan) compared to the manual segmentation (red), overlayed on the relative coronal CT slice. Images in each row come from a single scan progressing cranio-caudally from left to right.

DICE and Jaccard index increased (*P* < 0.001, both), while *ASSD* decreased (*P* = 0.003) and BF-score did not differ (*P* = 0.917) for *u*2*Net*_*Transfer*_ compared to *u*2*Net*_*Human*_. Absolute slopes on similarity *S*_*DICE*_ and *S*_*JI*_ increased while slopes on contour agreement measures did not differ *S*_*BF*_ and *S*_*ASSD*_ (Figure 3 and Table 3). Three slices in caudal to cranial sequence for representative scans of the *u*2*Net*_*Transfer*_ segmentations are shown in Figure 4.

**Table 3:** Segmentation quality metrics for the two networks trained on human data only *u*2*Net*_*Human*_ and on both experimental and clinical data sequentially *u*2*Net*_*Transfer*_.

	***u*2*Net*_*Human*_**	***u*2*Net*_*Transfer*_**	**Sign**.
*DICE* (arb. un.)	0.937 [0.93..0.95]	0.957 [0.94..0.97]	*P* < 0.001
*JI* (arb. un.)	0.882 [0.86..0.91]	0.918 [0.88..0.94]	*P* < 0.001
*BF* (arb. un.)	0.965 [0.92..0.98]	0.964 [0.91..0.98]	*P* = 0.917
*ASSD* (mm)	1.678 [1.2..2.51]	1.493 [0.7..2.45]	*P* = 0.003
*S*_*DICE*_ (arb. un.)	−0.12 {−0.15..−0.08}	−0.19 {−0.22..−0.17}	
*S*_*JI*_ (arb. un.)	−0.2 {−0.26..−0.14}	−0.34 {−0.39..−0.29}	
*S*_*BF*_ (arb. un.)	−0.246 {−0.34..−0.16}	−0.303 {−0.39..−0.21}	
*S*_*ASSD*_ (*mm*)	4.011 {2.2..5.82}	6.257 {4.34..8.17}	

*Values as median [iqr] and slope {95% conf. int.} respectively; Sørensen–Dice coefficient (DICE), with Jaccard index (JI), BF-score (BF) and F-score (BF), and average symmetric surface distance (ASSD) as well as their respective slopes S_DICE_, S_JI_, S_BF_, and S_ASSD_; statistics according to Wilcoxon test*.

#### 3.2.1. Dependence on Diagnosis

**Figure 5:**
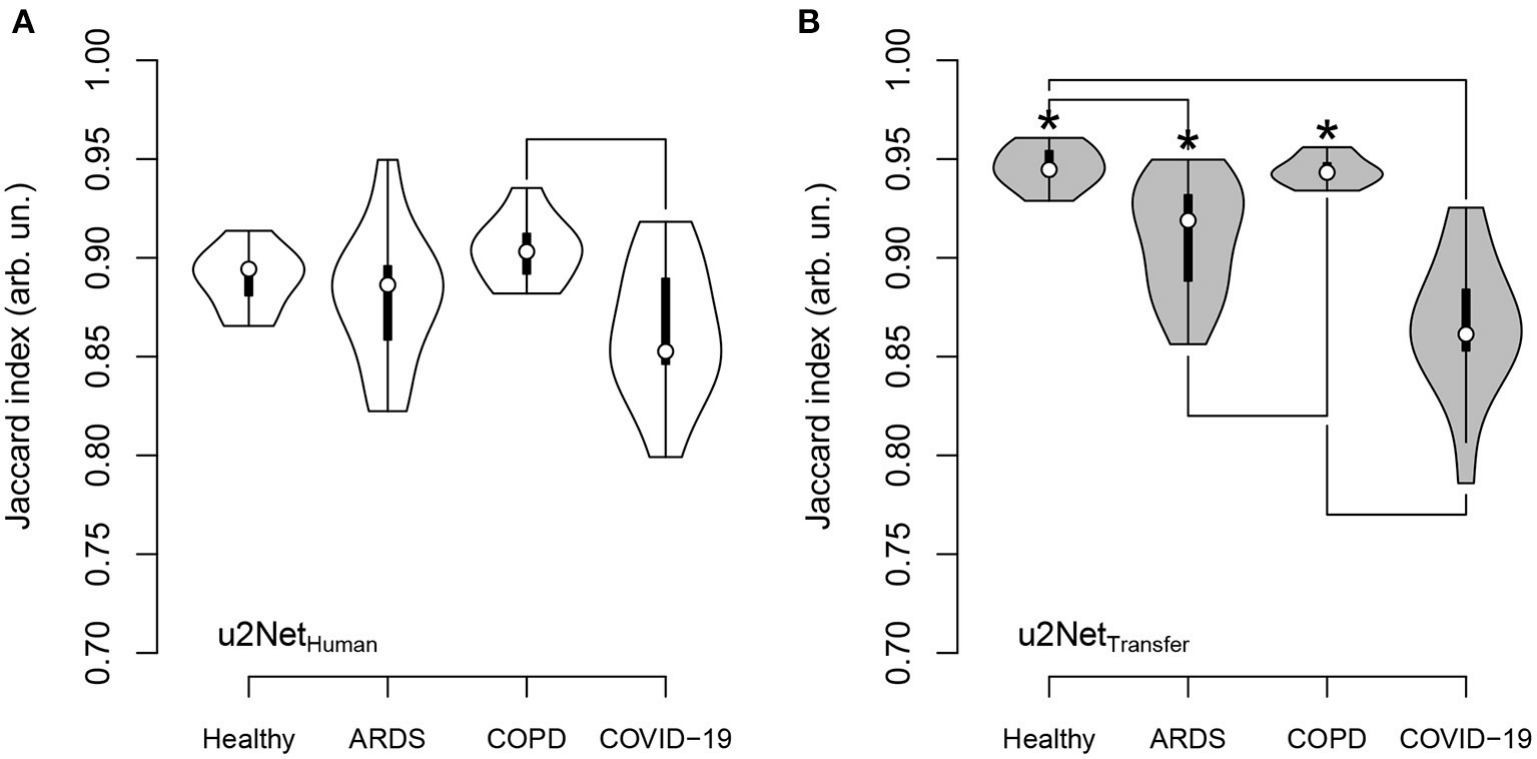
Jaccard index for *u*2*Net*_*Human*_ **(A)** and *u*2*Net*_*Transfer*_ **(B)** and its dependence on clinical diagnosis; significance was tested using Kruskal-Wallis test followed by Nemenyi test; asterisk (*) indicates difference between networks; lines between diagnosis indicate difference between diagnosis; significance accepted at *P* < 0.05.

The Jaccard Index computed from the predictions of *u*2*Net*_*Human*_ differed only between scans from COPD compared to COVID-19 patients (*P* = 0.006) in (Figure 5). Conversely the predictions of the network transfer learned *u*2*Net*_*Transfer*_ showed a significantly higher JI for scans of healthy lungs and COPD patients compared to scans from ARDS (*P* < 0.05) and COVID-19 patients (*P* < 0.05). Additionally, Jaccard Index was higher for all diagnosis except COVID-19 in the *u*2*Net*_*Transfer*_ vs. *u*2*Net*_*Human*_ networks. The total volume of the lung ROI determined by *u*2*Net*_*Transfer*_ differed from that determined through manual segmentation by 3.1 ± 189.5 *ml* (Supplementary Figure 4).

#### 3.2.2. Aeration Compartments

**Figure 6:**
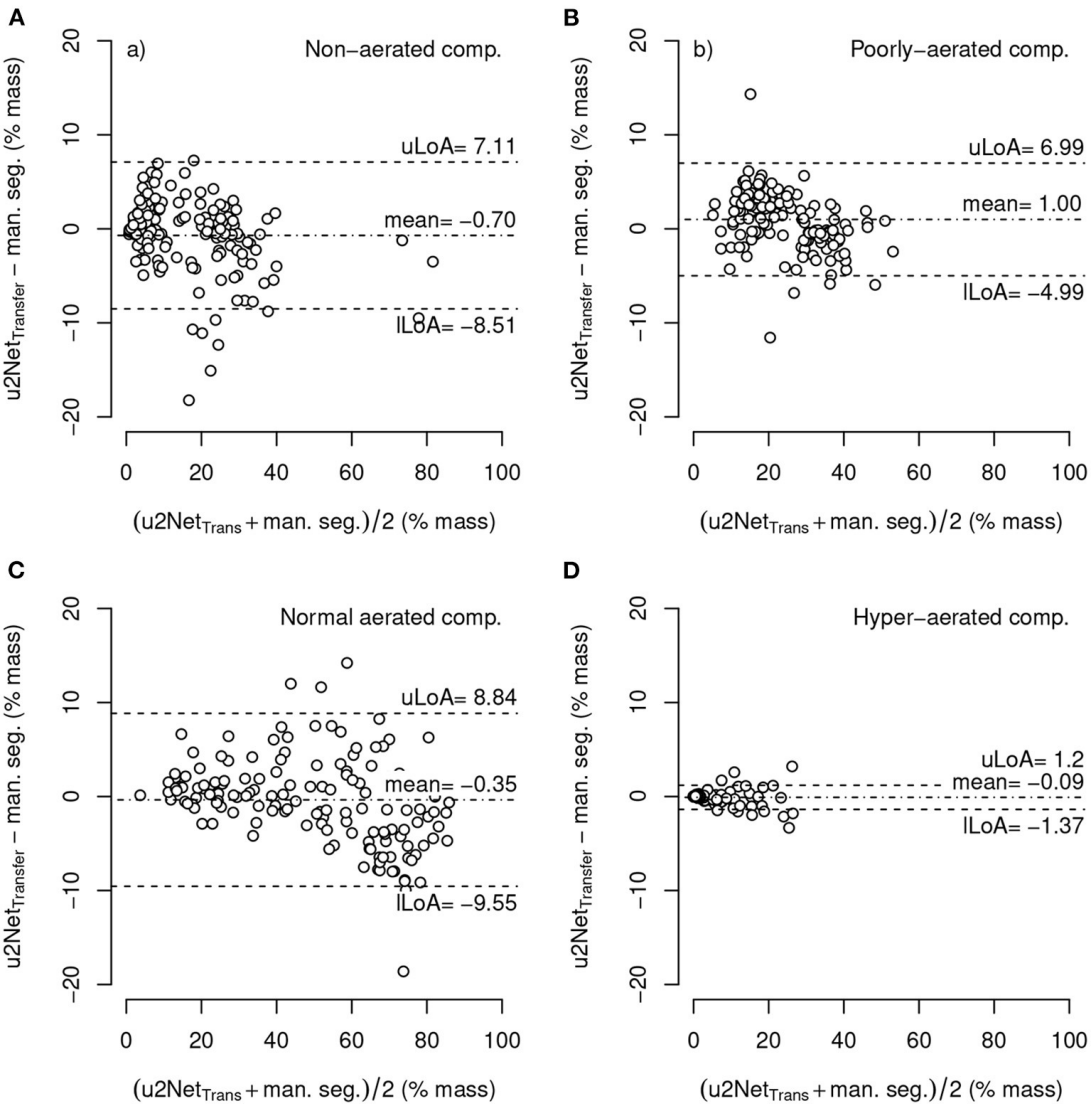
Bland-Altman-plot of relative mass non-aerated **(A)**, poorly-aerated **(B)**, normally-aerated **(C)** and hyper-aerated **(D)** compartments using mask segmented by *u*2*Net*_*Transfer*_ compared to manual segmentations; with upper and lower limits of agreement (mean ±1.96·standard deviation) *uLoA* and *lLoA*, respectively.

The relative mass of hyper-aerated lung regions as determined by the *uNet*2_*Transfer*_ segmentations had the smallest mean difference compared to that obtained through manual segmentation (−0.09 ± 0.66 *%mass*, *LoA* − 1.37 : 1.2) followed by normally- (−0.35 ± 4.69 *%mass*, *LoA* − 9.55 : 8.84), non- (−0.77 ± 3.98 *%mass*, *LoA* − 8.51 : 7.11), and poorly-aerated compartments (1.00 ± 3.06 *%mass*, *LoA* − 4.99 : 6.99), respectively (Figure 6). Independent of the compartment the Limits of agreement of the difference between both methods was well below 10%. For statistics on the relative volume of each aeration compartment refer to Supplementary Figure 3.

**Figure 7:**
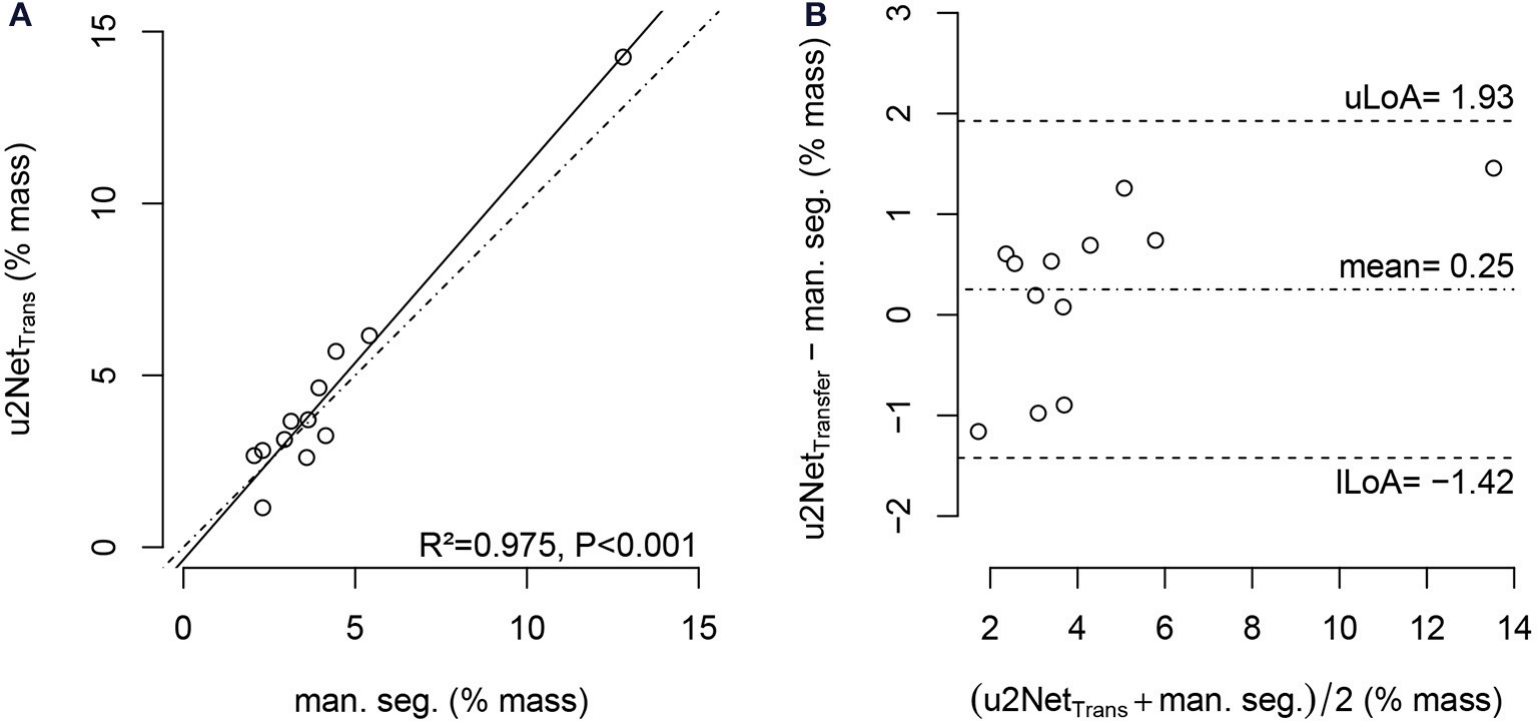
Regression plot **(A)** and Bland-Altman-analysis **(B)** of relative non-aerated and poorly aerated lung mass from *PEEP* = 16*cmH*_2_*O* to *PEEP* = 8*cmH*_2_*O* in COVID-19 scans using manual segmentation and automatic segmentation from *u*2*Net*_*Transfer*_; with upper and lower limit of agreement (mean ±1.96· standard deviation) *uLoA* and *lLoA*, respectively.

The relative mass of non- and poorly aerated compartments increased from *PEEP* = 16 *cmH*_2_*O* to *PEEP* = 8 *cmH*_2_*O*. The value determined using the *u*2*Net*_*Transfer*_ segmentation was highly correlated with the value obtained via manual segmentation (Figure 7) with limits of agreement below 2%.

#### 3.2.3. Effective Lung Volume

**Figure 8:**
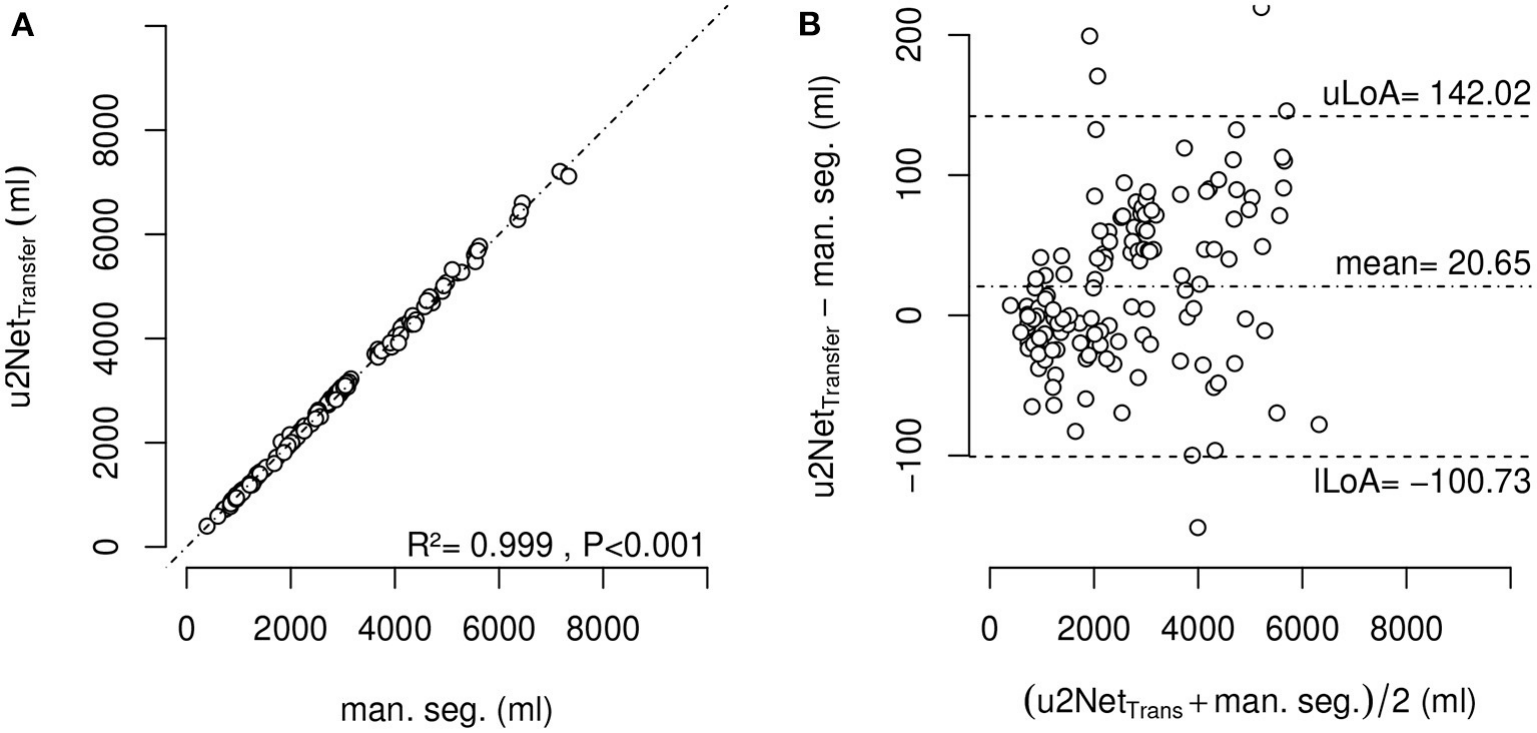
Effective lung volume (ELV) measured using segmentation by *u*2*Net*_*Transfer*_ over ELV measured using manual segmentation **(A)** and corresponding Bland-Altman-Analysis **(B)**; with upper and lower limit of agreement (mean ±1.96·standard deviation) *uLoA* and *lLoA*, respectively.

The determination of effective lung volume using *u*2*Net*_*Transfer*_ automated segmentation showed a difference with LV obtained through manual segmentations of 20.6 ± 61.9 *ml* (Figure 8). Additionally, total lung volume determination by automated and manual segmentations may be found in Supplementary Figure 4.

### 3.3. Computational Time

The proposed segmentation algorithm was tested on a commercially available personal computer equipped with an Intel i5 CPU and 8 *GB* of RAM. On this system, the algorithm could output low-resolution lung segmentation in under 20 s and a full resolution analysis in approximately 15 min.

## 4. Discussion

The main findings of this investigation can be summarized as follows. We developed and evaluated a three-dimensional U-net based algorithm for time-efficient segmentation of the lung parenchyma. The algorithm, consisting of two deep networks concatenated in series, yielded satisfactory performance, sufficient for potential clinical applications using quantitative non-aerated compartment volumetry. Training the network using transfer learning across species improved the segmentation quality on the Human data sets in all patient groups except COVID-19. The sizes of the aeration compartments and the effective lung volume could be determined with limits of agreement of 5% with manual segmentation. The analyses assessing the dependence of the Jaccard index and the BF-score on the relative non-aerated lung volume (*S*_*JI*_ and *S*_*BF*_, respectively) revealed that our proposed algorithm is able to perform robust segmentation of the diseased lungs.

The sub-analysis of lung recruitability shown in Figure 7 from a subset of patients with available manual segmentations at *PEEP* = 8 *cmH*_2_*O* and *PEEP* = 16 *cmH*_2_*O* shows a strong correlation between the two methods (*R*^2^ = 0.975). This, combined with the near-perfect correlation in determining ELV (*R*^2^ = 0.999) shown in Figure 8 and the Bland-Altman analysis of aeration compartments shown in Figure 6, suggests that our proposed approach is sufficient for the task of monitoring modifications of poor and non-aerated lung tissue.

The results presented here demonstrate that AI-based analysis of CT scans yield fast and efficient evaluations of lung aeration compartments. Such algorithms should therefore be tested more widely, especially given the potential benefits of the derived parameters to the management of ventilatory strategies in ARDS. The varying performance of the algorithms in different pathological conditions reflects the anatomical alteration of the healthy lung, an intrinsic property of lung pathologies. In COPD, emphysema will enhance HU difference between parenchyma and surrounding structures, while in ARDS (and especially COVID-19) consolidated lung regions have intrinsically difficult boundaries to identify on CT scans, even for human experts. Upon visual examination of the worst scan as shown in Figure 4 our algorithm is able to identify even the completely collapsed parenchyma, albeit with some uncertainty. This highlights the need, in developing data-driven approaches, for databases that span all required pathological conditions. The degree of detail that can be expected also suggests that this approach is suited for gross delineation of lung volumes and further research is needed to develop a system capable of finer distinction of blood vessels and airways.

One of the strengths of this technique is that it is operator-independent and highly reproducible. More importantly, if coupled with a simple threshold-based algorithm for identifying lung aeration compartments, this method can be used to quantify the degree of atelectasis or hyper-distension of lung parenchyma. The aforementioned qualities of AI-based analysis also reduce the cost of analyzing repeated CT scans, making it possible to follow the trend of pathological modifications over time and evaluate the effectiveness of interventions for both research and clinical purposes.

Quantitative analysis of aeration compartments could thus be implemented in decision making algorithms and contribute to the standardization of treatment across different settings and intensive-care units. The efficiency and accuracy of this method are appropriate for analysis of large data sets for research on lung disease that have until now been difficult to access.

This method may have potential clinical applications. While currently tidal volume is usually titrated to predicted body-weight, this method allows easy access to an estimation of lung tissue available for ventilation and can contribute to further development of lung protective strategies. Moreover, if coupled with dual-PEEP CT scans, it allows for an estimation of recruitability of the lung and can aid the clinician in the decision for recruitment maneuvers and PEEP setting. Finally, the quantification of non-aerated lung parenchyma could also be used to stratify severity and inform prognosis in ARDS.

The proposed transfer learned algorithm showed a lower performance compared to SegNet based LungSeg algorithm (*DICE* = 0.96 compared to *DICE* = 0.98) (Gerard et al., 2021) which may be explained by the lower number of available scans, the more heterogeneous diagnosis, and larger non-aerated relative lung regions in the data set.

Performance of the algorithm presented here was similar to the 3D uNet-based approaches trained on COVID-19 scans only with *DICE* = 0.96 (Müller et al., 2020). Although the latter had a better performance compared to the results on COVID-19 scans presented here (*DICE* = 0.93), it may be anticipated that the algorithm presented here may perform better on non-COVID ICU thorax CTs. Compared to 2D-uNet algorithms, our results indicate a slightly better performance on lung healthy patients (*DICE* = 0.95 vs. *DICE* = 0.97) (Ait Skourt et al., 2018) and outperformed results on COVID-19 patients (Zhou et al., 2021) (*DICE* = 0.83).

In evaluating the performance of lung segmentation algorithms in ARDS, we advocate for the use of a metric that takes into account the degree of non-aerated lung parenchyma present in the training and validation data sets. To this end, we propose a straight-forward slope index based on Jaccard and BF metrics. Low slopes in the experimental data set, compared to the human data set, suggest higher robustness toward non-aerated lung regions in the experimental data set, that might be explained by a more homogeneous nature of the surfactant depleted models. In the human data set both slope measures showed higher absolute value potentially due to the origin of non-aerated lung regions being more diverse and thus more heterogeneously distributed. This idea is supported by the fact that the transfer trained network indeed showed an increased Jaccard slope, compared to the network only trained on clinical data. A similar performance criterion had been implemented by Gerard et al. (2021) using the slope of DICE and ASSD with respect to relative volume of non-aerated lung compartments. Recalculation of the *S*_*DICE*_ in arb. un. to DICE slope in %^−1^ yielded a value of −0.0012 %^−1^ for the human only trained algorithm and −0.0019 %^−1^ for the transfer trained algorithm, both values being lower than the lowest value 0.003 %^−1^ reported by Gerard et al. (2021). Our algorithm trained on human data sets only showed lower ASSD slope with 0.04 *mm%*^−1^ compared to the one by Gerard et al. (2021) (0.07 *mm%*^−1^), while the transfer learned algorithm showed similar values 0.06 *mm%*^−1^.

This study has several limitations. Firstly, the training and evaluation were performed on scans from a relatively low number of distinct animals/patients using five-fold cross validation. While our results are in keeping with others previously published, it is likely that training our proposed system on larger data sets would yield better results. Secondly, animal data were taken only from experimental models of reversible atelectasis, not resembling heterogeneity and underlying cause of clinical ARDS. Thirdly, scans from different computed tomographic scanners, with different resolutions and kernels, were used for the applied lung volumetry. While this implies reduced comparability between the respective scans (Mascalchi et al., 2017), it may also be regarded as an advantage since the networks experienced a higher diversity during training and may therefore show higher performance during clinically diverse CT scan modalities (Hofmanninger et al., 2020). Fourthly, the data used for this investigations did only contain one manual segmentation for each CT scan. A comparison of the algorithm to inter-human manual segmentations could therefor not be performed. Finally, the deep learning convolutional neural network based approach consisting of two sequential networks had been proposed before (Gerard et al., 2021). The present manuscript describes a re-implementation in Matlab Deep Learning Toolbox trained and bench-marked on a limited data set focused on pathological lung segmentation in moderate ARDS where transferability between species was accounted for.

## 5. Conclusion

Automatic uNet based 3*D* lung segmentation showed good quality and thereby allowed reliable estimation of lung volumes, aeration compartment sizes, and lung recruitability in both animals and patients with different lung conditions.

## Data Availability Statement

The original contributions presented in the study are included in the article/Supplementary Material, further inquiries can be directed to the corresponding author.

## Ethics Statement

The studies involving human participants were reviewed and approved by Comitato Etico Regione Liguria, Italy. The need for written informed consent was waived for retrospectively collected data. According to local regulations, consent was delayed after discharge for prospectively collected data in unconscious patients. The animal study was reviewed and approved by Institutional Animal Care and Welfare Committee of the State of Saxony, Germany.

## Author Contributions

LM and RH designed and developed the algorithm and performed the training and evaluation of the algorithm. MM, FI, R-TH, and LB performed the manual segmentation of the clinical data set. LM, LB, NS, PP, MG, and RH designed the investigation and analysis protocol. All authors drafted, corrected, and revised the original manuscript.

## Funding

This work was made possible by institutional funds and in part by German Research Foundation (grant no. GA 1256/8-1).

## Conflict of Interest

The authors declare that the research was conducted in the absence of any commercial or financial relationships that could be construed as a potential conflict of interest.

## Publisher's Note

All claims expressed in this article are solely those of the authors and do not necessarily represent those of their affiliated organizations, or those of the publisher, the editors and the reviewers. Any product that may be evaluated in this article, or claim that may be made by its manufacturer, is not guaranteed or endorsed by the publisher.
