# Supplementary material for: Automatic Lung Segmentation and Quantification of Aeration in Computed Tomography of the Chest Using 3D Transfer Learning
Source: Front Physiol. 2022 Feb 4;12:725865. doi: 10.3389/fphys.2021.725865 (PMC8854801; doi:10.3389/fphys.2021.725865)
Supplement: Supplementary file 1 [file Data_Sheet_1.pdf]

Supplementary Material

Automatic lung segmentation and quantification of aeration in computed tomography of the chest using 3D transfer learning

Lorenzo Maiello [1,2,*], Lorenzo Ball [2], Marco Micali [2], Francesca Iannuzzi [2], Nico Scherf [3], Ralf-Thorsten Hoffmann [4], Marcelo Gama de Abreu [1,5], Paolo Pelosi [2] and Robert Huhle [1,*]

[1] Pulmonary Engineering Group, Department of Anaesthesiology and Intensive Care Therapy, University Hospital Carl Gustav Carus, Technische Universität Dresden, Dresden, Germany
[2] University of Genoa, Department of Surgical Sciences and Integrated Diagnostics, IRCCS AOU San Martino IST, 16131 Genoa, Italy
[3] Max Planck Institute for Human Cognitive and Brain Sciences, Leipzig, Germany
[4] Department of Diagnostic and Interventional Radiology, University Hospital Carl Gustav Dresden, Technische Universität Dresden, Dresden, Germany
[5] Department of Intensive Care and Resuscitation & Department of Outcomes Research, Anesthesiology Institute, Cleveland Clinic, Cleveland, Ohio, USA

Table of Contents

1. Data set details
2. Quality Measures for Comparing Segmentation
3. Relative volume of aeration compartments
4. Total lung volume

1 DATA SET DETAILS

Table S1. Patient specific and CT acquisition data for the various retrospectively analysed studies.

	Study			
	BIPAP	NEAP	KERNEL	PEEP
Reference	[1■]	n.a.	[2■]	[3■]
Species	pigs (german land race)		human	
Gender (f/m)	$11 \vert 0$	$7 \vert 0$	$16 \vert 24$	$4 \vert 14$
Body weight (kg)	$26 - 40$	$28 - 58$	n.a.	$61 - 73$
Age (years)	n.a. (juvenile)		$22 - 87$	$58 - 67$
Model/Diagnosis	repetitive lung lavage / Mild ARDS	lung healthy	lung healthy (10), COPD (10), ARDS(20)	mild/severe hypoxia and COVID-19
$V_{nA}(\%vol)$ median [min..max]	9.1 $[3.2..23.4]$	9.8 $[3.1..66.0]$	1.6 $[0.2..60.8]$	14.0 $[4.5..30.0]$
Kernel	BF70f	B30f	various	B60f
Number of subjects	11	7	40	18
	$\sum = 18$		$\sum = 58$	
Number of scans	68	112	106	44
	$\sum = 180$		$\sum = 150$	

2 QUALITY MEASURES FOR COMPARING SEGMENTATIONS

This section provides the comparison of different measures of similarity and distance during mono-class segmentation in a simple numerical model assuming a spherical reference segmentation Ref with a radius of $r = 40$ full-filling

$$(x - x_m)^2 + (y - y_m)^2 + (z - z_m)^2 \leq r^2,$$

and an assumed segmentation under investigation Seg of any algorithm as uni-axial and oblate/prolate ellipsoids according to

$$\frac{(x - x_m)^2}{a^2} + \frac{(y - y_m)^2}{r^2} + \frac{(z - z_m)^2}{r^2} \leq 1$$

with radius of elliptic dimension a with-in 0 to 80 voxel as depicted in the insets of Fig. S1.

Comparison between measures of set difference Dice score and Jaccard index over elliptic dimension a of Seg (Fig. S1), and Hausdorff distance, $ASSD$ and BF-score (Fig. S2). Jaccard index was chosen as a measure of similarity since it adds more granularity due to its steeper, more linear dependence on the deviation during these experiments. Similarly, it was opted for BF-score in favour of ASSD and Hausdorff distance with in this manuscript. However the other measures are given for completeness.

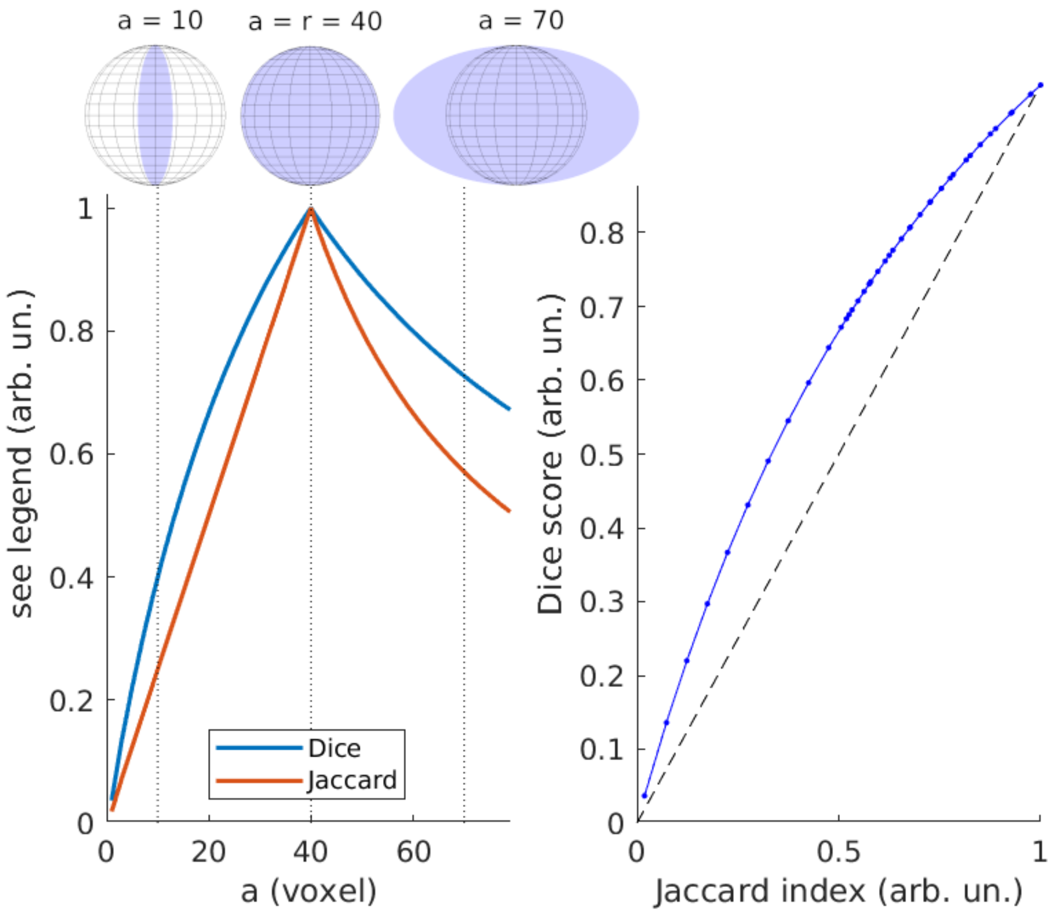

Figure S1. DICE score and Jaccard index over radius of elliptic dimension a (left) and relationship between DICE and Jaccard index (right) during described simulation with a spherical reference segmentation (black grid, $r = 40$) and different segmentations under test (blue,insets).

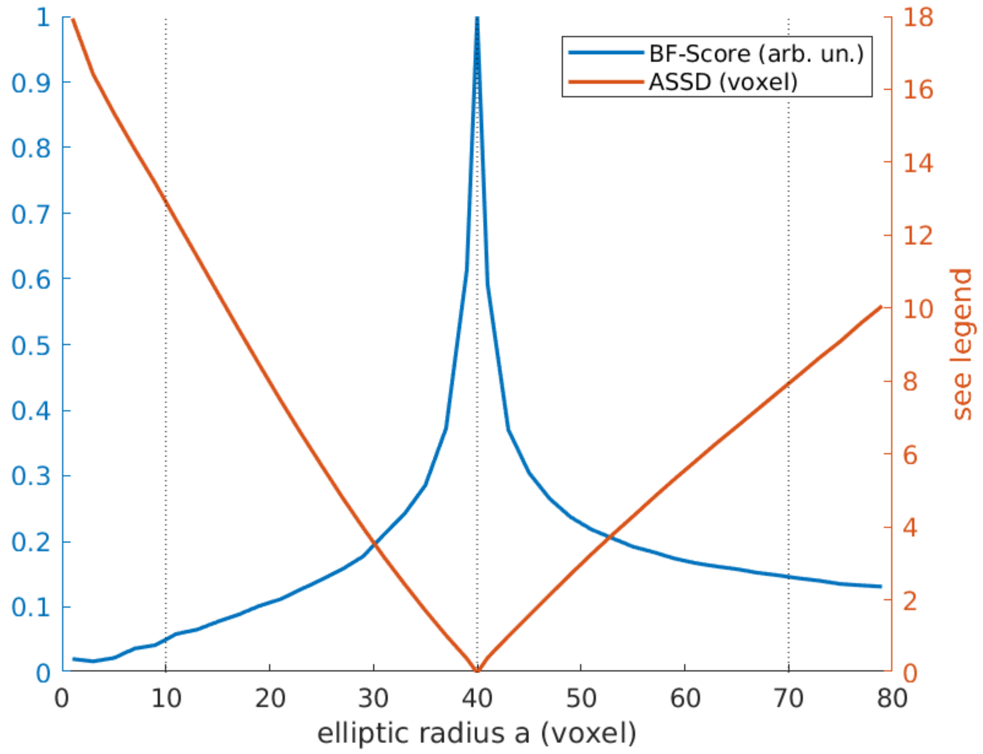

Figure S2. BF-score and average symmetric surface distance ($ASSD$) over radius of elliptic dimension a during described simulation with a spherical reference segmentation (black grid, $r = 40$) and different segmentations under test (blue,insets), for details see text and Figure S1.

3 RELATIVE VOLUME OF AERATION COMPARTMENTS

Relative volume of hyper-aerated lung regions as determined using the $u2Net_{Transfer}$ segmentations had the smallest mean difference compared to expert manual segmentation ($0.02 \pm 0.51\ \%vol$) followed by poorly- ($0.21 \pm 1.42\ \%vol$), normally- ($0.25 \pm 2.50\ \%vol$) and non-aerated compartments ($-0.50 \pm 2.14\ \%vol$), respectively (Fig. S3). Independent of the compartment the limits of agreement of the difference between both methods was below $5\ \%volume$ and $10\ \%mass$.

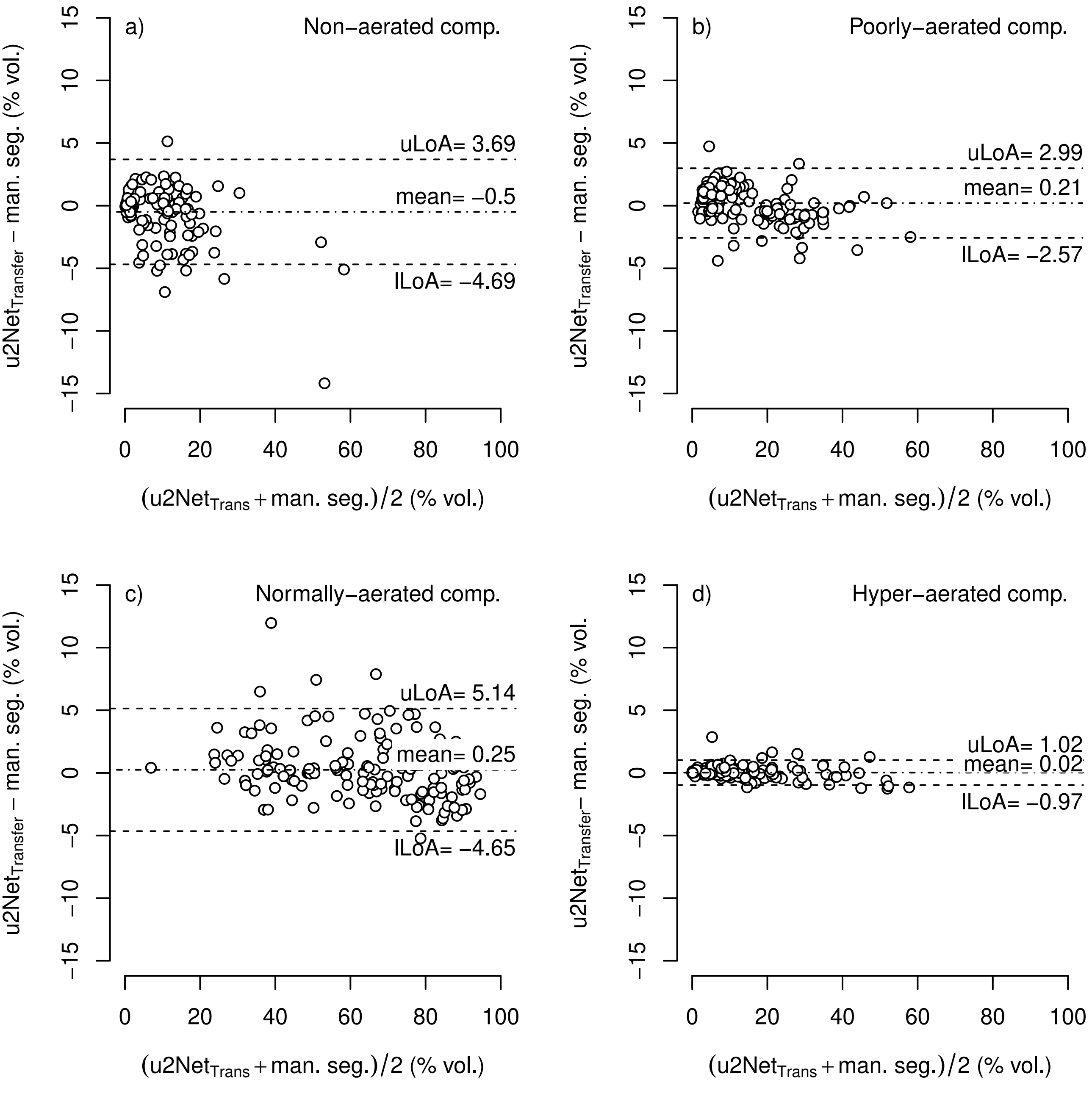

Figure S3. Bland-Altman-Plot of relative volume of non-aerated a), poorly-aerated b), normally-aerated c) and hyper-aerated d) compartments using mask segmented by $u2Net_{Transfer}$ compared to manual segmentations; with upper and lower limits of agreement (mean$\pm 1.96\cdot$standard deviation) $uLoA$ and $lLoA$, respectively.

4 TOTAL LUNG VOLUME

Volume of Lung ROI from $uNet2_{Trans}$ differed from expert reference by $-46.4 \pm 208.0\ ml$.

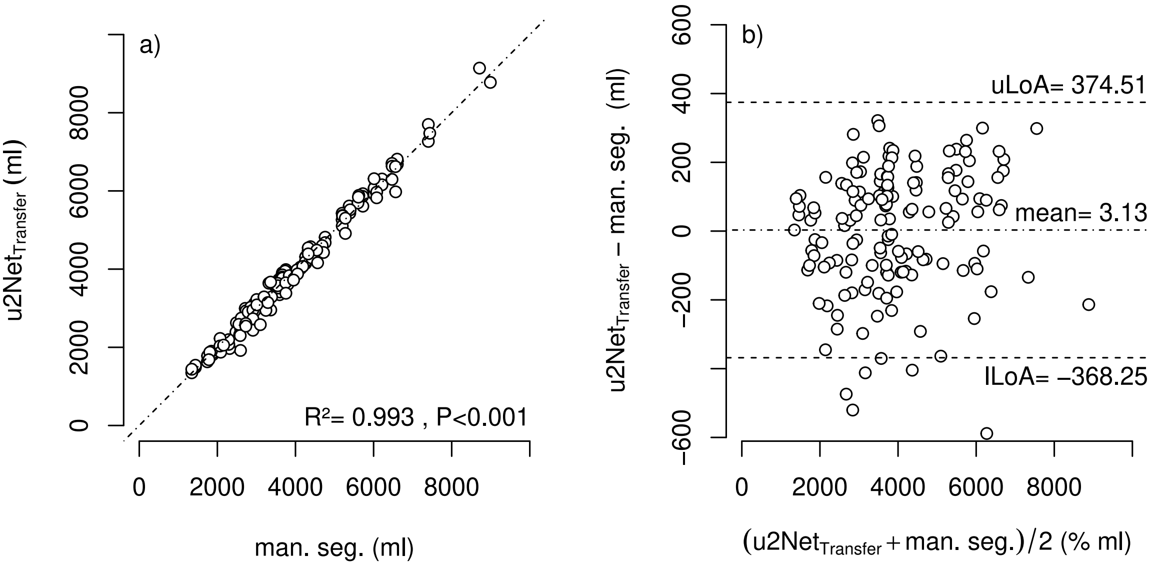

Figure S4. Size of lung ROI segmentation by transfer learned neural network $uNet2_{Trans}$ vs. manual reference segmentation.

REFERENCES

[1■]Andreas Güldner et al. “Higher Levels of Spontaneous Breathing Induce Lung Recruitment and Reduce Global Stress/Strain in Experimental Lung Injury”. eng. In: *Anesthesiology* 120.3 (Mar. 2014) pp. 673–682. ISSN: 1528-1175. DOI: `10.1097/ALN.0000000000000124`.

[2■]Lorenzo Ball et al. “Lung Hyperaeration Assessment by Computed Tomography: Correction of Reconstruction-Induced Bias”. eng. In: *BMC Anesthesiol* 16.1 (Aug. 2016) p. 67. ISSN: 1471-2253. DOI: `10.1186/s12871-016-0232-z`.

[3■]Lorenzo Ball et al. “Computed Tomography Assessment of PEEP-Induced Alveolar Recruitment in Patients with Severe COVID-19 Pneumonia”. In: *Critical Care* 25.1 (Feb. 2021) p. 81. ISSN: 1364-8535. DOI: `10.1186/s13054-021-03477-w`.
